# The impact of generalized joint laxity on clinical outcomes of total knee arthroplasty

**DOI:** 10.1007/s00167-017-4486-x

**Published:** 2017-03-06

**Authors:** Sae Kwang Kwon, Hyuck Min Kwon, Youngho Kong, Kwan Kyu Park

**Affiliations:** 1 Department of Orthopedic Surgery, Yonsei Sarang Hospital, Bucheon, South Korea; 2 Department of Orthopedic Surgery, Yonsei University College of Medicine, 50-1 Yonsei-ro, Seodaemun-Gu, Seoul, 120-752 South Korea

**Keywords:** Total knee arthroplasty, Generalized joint laxity, Clinical outcome, Degenerative osteoarthritis

## Abstract

**Purpose:**

The aim of this study was to investigate whether the severity of generalized joint laxity influences preoperative and postoperative clinical outcomes and if patients with severe generalized joint laxity would require a thicker polyethylene (PE) liner during total knee arthroplasty (TKA).

**Methods:**

A total of 338 female patients undergoing TKA were divided into two groups according to generalized joint laxity. Preoperative and postoperative (at 3 years) patellofemoral scale, AKS, WOMAC, ROM, and satisfaction VAS were compared between the two groups. Additionally, PE liner thickness was compared.

**Results:**

Preoperatively, flexion contracture and WOMAC stiffness scores in the severe laxity group were significantly lower than those in the no to moderate laxity group (*p* < 0.001 for both). There was no significant difference in postoperative clinical outcomes of patellofemoral scale, AKS, WOMAC, or ROM or in satisfaction VAS between the two groups. There was a significant difference in PE liner thickness between the two groups (10.3 ± 1.3 versus 11.4 ± 1.2, *p* = 0.043).

**Conclusions:**

There was no significant difference of clinical outcomes between the patients with and without severe generalized joint laxity after 3 years of follow-up after TKA, even though preoperative clinical outcomes indicated that the patients with severe generalized joint laxity showed significantly smaller flexion contraction and better WOMAC stiffness score. Since patients with generalized joint laxity require a thicker PE liner, care should be taken to avoid cutting too much bone from patients with severe generalized joint laxity.

**Level of evidence:**

Retrospective comparative study, Level III.

## Introduction

Little is known about the effects of patient’s soft tissue physiology on the outcomes of total knee arthroplasty (TKA). Both bone resection and a procedure that manipulates the soft tissue surrounding the knee are important to correct alignment and adjust the gap in TKAs. Therefore, the patient’s soft tissue physiology could be a critical factor that impacts surgical outcomes of TKA. Generalized joint laxity, a part of soft tissue physiological factor, would be an important factor that can impact TKA outcomes.

Several studies have reported that generalized joint laxity is a risk factor for poor clinical outcomes of soft tissue reconstruction surgeries [17, 23]. Especially, it is widely known that surgical outcomes of anterior cruciate ligament (ACL) injury is associated with the generalized joint laxity [18–20]. Although the soft tissue is important in TKAs and is a critical factor of surgical outcome, generalized joint laxity has never been studied in TKA.

Therefore, the purpose of this study was to investigate whether the severity of generalized joint laxity influences preoperative and postoperative clinical outcomes in addition to polyethylene (PE) liner thickness in TKA. The hypotheses of the current study were that patients with severe generalized joint laxity would have a larger range of motion than patients with no to mild generalized joint laxity, both preoperatively and postoperatively. Moreover, we postulated that patients with severe generalized joint laxity would require a thicker PE liner during TKA, using a measured resection technique, than those with no to mild generalized joint laxity, since the ligaments of patients with severe generalized joint laxity would be attenuated.

## Materials and methods

### Patient recruitment and generalized joint laxity measurements

Three hundred thirty-eight patients that underwent unilateral knee replacement between March 2009 and February 2011 and that completed 3 years of follow-up were included in this study. Patients with lower extremity deformities, such as severe varus or valgus knee deformity, which needs osteotomy together with TKA or special implants, such as varus or valgus constraint, previous surgery, secondary osteoarthritis, or inflammatory osteoarthritis, including rheumatoid arthritis, were not included in this study. All of the recruited patients were female, and the median age was 68 years (range 58–81), with a median BMI of 25.9 kg/m^2^ (range 19.8–31.2).

The Beighton and Horan criteria were used by an independent investigator to evaluate generalized joint laxity and clinical outcomes of all patients during the study period, prior to surgery [2]. (1) passive dorsiflexion of the little fingers; (2) passive apposition of the thumbs; (3) hyperextension of the elbows; (4) hyperextension of the knees; and (5) forward flexion of the trunk. All elements were added to obtain an overall joint laxity score that ranged from 0 (normal) to 5 (hyperlaxity). The patients were divided into two groups: group A was a ‘none to moderate laxity group’ that met 0 to 3 elements of the scale (284 patients), and group B was a ‘severe laxity group’ that met 4 or 5 elements of the scale (54 patients) (Table 1).

**Table 1:** Patient demographics

Generalized joint laxity scores	No. of patients (%) (*n* = 338)	Age (year)	BMI (kg/m^2^)
0	90 (26.6%)	68.2 ± 3.8	26.4 ± 3.8
1	72 (21.3%)	68.8 ± 3.8	25.3 ± 3.3
2	53 (15.7%)	66.7 ± 3.8	26.2 ± 3.6
3	69 (20.4%)	68.0 ± 3.8	26.1 ± 3.4
4	45 (13.3%)	68.0 ± 3.8	26.2 ± 3.7
5	9 (2.7%)	66.7 ± 3.8	27.2 ± 4.2
Mean ± SD		69.9 ± 7.4	26.1 ± 3.6
*p* value		ns	ns

Data are presented as number (%) or mean ± standard deviation

*BMI* body mass index

### TKA procedure and clinical outcome evaluation

All procedures were performed by a single surgeon using the standard medial patellar approach with tourniquet inflation and the measured resection technique including selective soft tissue release. For the distal femoral cut, we performed a posterior capsular release of the distal femur and removed osteophytes when there was flexion contracture. When the flexion contracture remained after these procedures, we performed an additional distal femur cut; this additional distal femur cut was not routinely performed for patients with flexion contracture. A posteriorly stabilized prosthesis (PFC, Depuy, IN, USA) was implanted in all cases. The patella was resurfaced in all cases, and cement fixation was used for all components. The polyethylene (PE) liner thickness was measured to compare between the two groups.

All clinical information was collected using pre-designed datasheets in the out-patient clinic preoperatively and postoperatively and maintained in our database by an independent investigator. The clinical information included demographic data, preoperative clinical status, and postoperative outcomes. The preoperative clinical status and postoperative outcomes were evaluated based on the following: the motion arc of the knee, the American Knee Society (AKS) score [12], the Western Ontario McMaster University Osteoarthritis Index scale score (WOMAC) [3, 4, 11], and the Kujala patellofemoral scale score [21].

The motion arc of the knee was represented by maximum flexion and range of motion (ROM), which was calculated by subtracting the degree of flexion contracture from the degree of maximum flexion. An independent investigator used a goniometer to measure flexion contracture and maximum flexion to the nearest 5°, with the patient in the supine position. Two measurements of each patient were made by one surgeon. The degree of measurement reliability was assessed using intraclass correlation coefficients. The 95% confidence intervals of intraclass correlation coefficients were 0.941–0.968. Patient satisfaction was evaluated using the visual analog scale (VAS), which is graded from 0 to 10 (10, best). The data collection method and research design were approved by the Institutional Review Board (IRB) of Severance Hospital (IRB # 4-2015-0468).

### Statistical analysis

Sample size calculation was performed to detect a significant difference in WOMAC score, which has been widely used for evaluation of clinical outcomes of TKA [3, 4]. The sample size calculation was based on WOMAC scores observed in a pilot study conducted at the author’s hospital that included 20 patients. A sample size of 53 patients in each group was required for a power of 80% at a type I error level of 0.05 and for an expected dropout rate of 20%. Post hoc power analysis for detecting differences in the measurement outcomes between the two groups was conducted with a significance level of 0.05 and an effect size of 0.3. There were 50 knees in each group; therefore, the power of this study was estimated to be 87.5% in the power analysis. The statistical software G*Power (Erdfelder, Faul, Buchner & Lang Behavior Research Methods, Instruments & Computers, Germany, 2014) was used for power analyses. Student’s *t* tests were used to compare the means of the variables of Group A (none to moderate laxity group) and Group B (severe laxity group). ANOVA was also used to compare the means of the variables among six groups with generalized joint laxity scores from 0 to 5. Statistical analyses were performed using SPSS software for Windows (Version 20.0, SPSS, Chicago, IL), and *p* values <0.05 were considered significant.

## Results

There was no significant difference in age or BMI between the groups divided according to generalized joint laxity score (Table 1).

All subjects experienced degenerative osteoarthritis, and the degree of degenerative osteoarthritis was Kellgren–Lawrence grade III or IV. Preoperative mean anatomical tibiofemoral alignment was varus 4.6° (±5.1) and postoperative mean anatomical tibiofemoral alignment was valgus 6.2° (±3.3). Preoperative clinical outcomes indicated that there was a significant difference in flexion contracture (4 ± 7 versus 1 ± 5 *p* < 0.001) and WOMAC stiffness score (3.2 ± 2.0 versus 2.4 ± 1.9, *p* < 0.001) between Group A (none to moderate laxity group) and Group B (severe laxity group) (Table 2). No other significant differences were found between the two groups (Table 2).

**Table 2:** Clinical outcomes based on generalized joint laxity severity

	Group A^a^ (*n* = 284)	Group B^a^ (*n* = 54)	*p* value
Preoperative clinical outcomes			
Flexion contracture	4° ± 7°	1° ± 5°	<0.001
Range of motion	124° ± 15°	128° ± 14°	0.014
AKS knee score	60.2 ± 14.4	60.1 ± 11.1	ns
AKS function score	46.8 ± 20.2	47.0 ± 19.0	ns
WOMAC (pain)	9.2 ± 3.0	9.3 ± 2.8	ns
WOMAC (stiffness)	3.2 ± 2.0	2.4 ± 1.9	<0.001
WOMAC (function)	33.0 ± 8.7	31.8 ± 7.2	ns
PF score	19.1 ± 5.8	18.0 ± 5.6	ns
Postoperative clinical outcomes (final follow-up of 3 years)			
Flexion contracture	1° ± 4°	1° ± 5°	ns
Range of motion	131° ± 11°	139° ± 9°	ns
AKS knee score	92.9 ± 6.8	92.6 ± 6.2	ns
AKS function score	72.6 ± 18.3	78.0 ± 16.6	ns
WOMAC (pain)	1.7 ± 2.3	1.5 ± 1.5	ns
WOMAC (stiffness)	1.6 ± 1.8	1.1 ± 1.4	ns
WOMAC (function)	16.9 ± 10.0	18.0 ± 7.8	ns
PF score	26.3 ± 4.0	25.4 ± 3.6	ns
VAS	8.8 ± 1.9	8.7 ± 1.7	ns
PE thickness (mm)	10.3 ± 1.1	11.4 ± 1.2	0.043

Data are presented as mean ± standard deviation

*AKS* American Knee Society, *WOMAC* Western Ontario and McMaster Universities, *PF* patellofemoral, *VAS* visual analog scale, *PE* polyethylene

^a^Group 1 was a ‘none to moderate laxity group’ that met 0 to 3 elements of the generalized joint laxity scale, and Group 2 was a ‘severe laxity group’ that met 4 or 5 elements of the scale

Postoperatively, there was no significant difference in the clinical outcomes of ROM, AKS, WOMAC, and VAS between the two groups postoperatively, including those at last follow-up of 3 years (Table 2). The mean value of the PE liner size was 10.7 ± 1.1 mm, and there was a significant difference in PE liner thickness between the two groups (10.3 ± 1.3 versus 11.4 ± 1.2, *p* = 0.043) (Table 2). There were no complications, such as dislocation, or revision surgeries due to infection or loosening during the 3 years of follow-up.

## Discussion

The most important finding of this study was that the severity of generalized joint laxity was not an influential factor on postoperative clinical outcomes of TKA, even though preoperative clinical outcomes were influenced by the severity of generalized joint laxity. This study demonstrated that patients with preoperative severe generalized joint laxity exhibit reduced flexion contracture, in addition to lower WOMAC stiffness score, compared to patients with no to moderate generalized joint laxity. Although patients with severe generalized joint laxity required a thicker PE insert, there was no significant difference in postoperative clinical outcomes between the two groups at 3-year follow-up. Contrary to soft tissue reconstruction surgeries, severe generalized joint laxity was not a risk factor for poor clinical outcomes of TKA. Therefore, surgeons do not need to hesitate to consider TKA on the patients with severe generalized joint laxity.

Previous studies have emphasized the importance of preoperative evaluation of soft tissue in TKA [16, 25]. Specifically, the soft tissue around the knee is important to evaluate to obtain good clinical outcomes [13, 30]. However, previous studies are limited to soft tissue laxity around the knees for assessing clinical outcomes of TKA [5, 15, 26–28]. This is the first study to assess the correlation between TKA and generalized joint laxity, which reflects natural physiological characteristics that are generalized joint laxity rather than localized laxity of soft tissue around knees.

Because all subjects experienced terminal stage degenerative osteoarthritis, there was no significant difference in AKS knee score, AKS function score, WOMAC pain score, or WOMAC function score between the two groups based on clinical scores before and after operation. The progression of degenerative osteoarthritis-induced pain due to ligament stiffness and capsular structure also caused a decrease in the ROM [8]. However, this study revealed that patients with severe generalized joint laxity have lower WOMAC stiffness scores and lower flexion contracture. This indicates that severe generalized joint laxity represents fewer symptoms related to stiffness and flexion contracture than none to mild generalized joint laxity. Because preoperative ROM preserves innate physiological characteristics, regardless of degenerative osteoarthritis progression to the terminal stage, patients with severe generalized joint laxity had greater ROM than patients with none to mild generalized joint laxity. Thicker inserts were needed for patients with severe generalized joint laxity in this study. The reason why the thicker inserts were needed with the patients with generalized joint laxity, compared to those without it, is uncertain. However, since surgeons often apply the same method or protocol for all TKAs, surgeons may need to take care not to cut too much bone if a patient has generalized joint laxity. For example, we assume that additional distal femoral cut would not be needed, even if there is a certain degree of flexion contracture with a patient with generalized joint laxity. Surgeons may consider further distal femoral cutting if there is a remaining flexion contracture, after checking the flexion–extension gap after routine bone cutting for a patient with generalized joint laxity.

Patellofemoral joint subluxation, which is induced by an increase in patellofemoral joint laxity, can cause additional knee symptoms related to patellofemoral alignment, such as anterior knee pain; this is based on studies that have identified a correlation between patellofemoral pain and generalized joint laxity [1, 6, 10, 29]. There was no significant difference in patellofemoral scale scores between the two groups after 3 years of follow-up or in other clinical outcomes in our study.

There were several limitations to this study. First, the follow-up period was relatively short. Even though the clinical outcomes plateaued 1 year after surgeries, and even if there were no significant differences in clinical outcomes between the two groups after up to 3 years of follow-up, it is possible that there are differences in the clinical results, including survival that could be identified with longer follow-up periods. Therefore, additional studies with longer follow-up are needed. Second, we did not evaluate the stress radiographies for anterior-posterior or medial–lateral laxity. Stress radiography has been used to evaluate patients with knee laxity [7, 9, 14, 22], but has not been generally used after TKA. Stress radiography could provide additional information to determine subtle differences, but we did not use this assessment in our clinical settings. Finally, we used only one type of implant (posterior substituting and fixed bearing type TKAs), and all patients were female. Using different insert types (cruciate retaining or mobile bearing type TKAs) of implants could have produced differing results because different insertions could affect knee stability. Further studies that can recommend different implant types according to the severity of the laxity would be helpful the surgeons. Additionally, results for male patients could also differ because generalized joint laxity can differ according to sex [24]. Female dominance in TKA is very typical in Asian countries; therefore, the results of this study should be carefully considered when applying to different types of implant or patient groups. Only primary TKA cases with or without generalized joint laxity were included in this study. Accordingly, a follow-up study with deformity cases that have joint instability with ligament attenuation is warranted in the future. Nevertheless, in primary TKA cases, severe generalized joint laxity was not a risk factor for poor clinical outcomes.

## Conclusion

In the current study, there was no significant difference in clinical outcomes between the patients with and without severe generalized joint laxity in our study after 3 years of follow-up TKA, even though preoperative clinical outcomes indicated that the patients with severe generalized laxity showed significantly smaller flexion contraction and better WOMAC stiffness score. Since the patients with generalized joint laxity needed a thicker PE liner, care should be taken to avoid cutting too much bone in patients with severe generalized joint laxity, especially when using a measured resection technique.
